# The Relationship Between Arterial Stiffness and Resistance Training

**DOI:** 10.7759/cureus.20213

**Published:** 2021-12-06

**Authors:** Ethan A Tabaie, Akshay J Reddy, Deeksha Mamidi, Nadine Khalil, Zeyu Yu, Gordon H Arakji, Hetal Brahmbhatt

**Affiliations:** 1 Neurobiology, Physiology and Behavior, University of California, Davis, Davis, USA; 2 Opthalmology, California Northstate University, College of Medicine, Elk Grove, USA; 3 Health Sciences, California Northstate University, Rancho Cordova, USA; 4 Psychiatry, Mercy General Hospital, Sacramento, USA

**Keywords:** cardiovascular disease, pulse wave, exercise, hypertension, resistance training, arterial stiffness

## Abstract

Exercise is a critical factor that impacts arterial stiffness. In this narrative review, we noted multiple findings that could not be reconciled with one another. Some studies indicated that arterial stiffness increases after a regimen of resistance training. However, such studies were limited by a lack of specification of the resistance training protocols, as well as varying results reported from different areas of the body, undermining the internal validity of the studies. Another factor explored in this review was how the order of performing exercises can affect arterial stiffness. Low-intensity resistance training before high-intensity resistance training resulted in increased arterial stiffness, whereas vice versa showed no change in arterial stiffness. Other studies indicated that resistance exercise results in reduced arterial stiffness. Intensity is a variable in studies that produces inconsistent results of arterial stiffness, with some studies suggesting high-intensity resistance training increases arterial stiffness and low-intensity resistance training decreases arterial stiffness, while other studies pointing to a significant decrease in arterial stiffness, regardless of the intensity of resistance training. Demographic factors such as gender, age, and diet play an important role in explaining these differences. In terms of future implications, there is potential clinical significance as increased arterial stiffness serves as a prognostic marker in diagnosing coronary heart disease.

## Introduction and background

It is widely known that cardiovascular diseases (CVDs) are the leading cause of mortality and morbidity in developing countries. In the United States alone, CVDs have led to the death of 600,000 individuals, which is approximately 25% of the total deaths [1]. Several studies have attempted to understand the factors that increase the risk of CVDs in individuals, particularly genetic factors, pathophysiology, and lifestyle choices. According to the literature, lack of physical activity and an unhealthy lifestyle are common factors that lead to poor cardiovascular health [2].

Exercise affects the cardiovascular system in a multitude of ways. Dynamic exercise results in an increase in heart rate, an increase in stroke volume, an increase in cardiac output, and a reduction in cholesterol [3]. There are key differences when considering diastolic and systolic blood pressure. Systolic blood pressure is proportionally related to the intensity of the exercise, and consequently, increases with bursts of activity. On the other hand, diastolic blood pressure remains either stable or lowers [4]. In addition, exercise increases the concentration of vascular endothelial growth factor (VEGF), leading to arteriogenesis in the myocardium through the recruitment of progenitor cells [4].

A loss of arterial stiffness is a major independent cause of common chronic cardiovascular diseases and increased morbidity and mortality. Arterial stiffness indicates the rigidity of the blood vessels around the heart. Low stiffness implies that there is low pressure exerted on the walls of the arteries or blood vessels during systole [5]. If the blood vessel walls become harder and stiffer, the heart works harder to pump more blood into the arteries as the pulse pressure widens. This increased stiffness can cause arterial clogging which can lead to heart attacks. Blood pressure and age influence arterial stiffness the most. To avoid an increase in blood pressure, individuals with stiffer arterial walls will only be able to utilize a small fraction of each cardiac stroke volume for blood circulation. Therefore, the blood pressure increases to make up for the abundant amount of blood in the peripheries. This results in lower diastolic pressure and higher pulse and systolic pressure.

Arterial stiffness is associated with changes in the mechanical properties of the arterial wall [4]. These arterial properties include the fractional deformation or strain and the applied force per unit of area or stress. The alterations in the fundamental mechanical behavior and properties of the arterial wall lead to a reduced ability to stretch properly [6]. It has been recognized that central arterial stiffening, a consequence of aging, can significantly contribute to CVD in older individuals by affecting the vascular phenotypes [7]. Peripheral arterial stiffening is associated with an increase in arterial stiffness, systolic hypertension, and disturbed wave reflection [8].

Several risks are associated with increased arterial stiffness. Increased arterial stiffness serves as a prognostic marker in diagnosing coronary heart disease [9]. Multiple studies have indicated that higher aortic stiffness (indicated through pulse wave velocity) is associated with an increased risk for a first cardiovascular event to occur [10,11].

Although it is commonly understood that resistance training increases arterial stiffness, there remains a gap in knowledge regarding the overall effects of resistance training on arterial stiffness. Thus, this review aims to analyze 23 randomized controlled trials to determine the relationship between full-body resistance training and arterial stiffness. Our efforts to understand the impact of resistance training on one of the major causes of CVDs can pave the way for future medical research and advancements.

## Review

Stiffness associated with large arteries is a proven risk factor for CVD [12]. Patients with end-stage renal failure and hypertension are even more likely to die when having arterial stiffness [13]. Individuals who perform aerobic training have lower arterial stiffness [14], and intense resistance training increases arterial stiffness [15]. However, several studies in the literature report a broader argument that resistance training increases arterial stiffness, which is not consistent with the current research. Several nuances were found regarding the effects of resistance training on arterial stiffness.

Significant increases in arterial stiffness

Within the review, out of the 23 randomized controlled trials analyzed, seven showed significant increases in arterial stiffness after a regimen of resistance training [16-22]. However, within this statement, there is still nuance. According to Collier et al., prehypertensive and hypertensive patients showed an increase in arterial stiffness after resistance training [16]. This indicates that hypertension may increase arterial stiffness or it may result in no change in arterial stiffness if arterial stiffness was solely increased by resistance training. This can be difficult to confirm because the type of resistance training was also shown to affect central pulse wave velocity values, which were used to measure arterial stiffness. Two studies reported an increase in arterial stiffness in both men and women only after concentric resistance training, where weight lifting was slow and weight lowering was quick [17,18]. However, the opposite lifting pattern of eccentric resistance training showed no significant change from the baseline [17,18]. Many of these studies may have compromised results because of the lack of specification of their resistance training protocols, which may be a reason for studies reporting no significant change in arterial stiffness. Another discrepancy was seen in arterial stiffness values. When performing resistance training in the upper limb, there was an increase in arterial stiffness, while lower limb resistance training showed no change in arterial stiffness [19]. This brings to question the reliability of many studies recording arterial stiffness values as even training different parts of the body showed varying results. Finally, one study tested the order of intensity of a resistance exercise and showed that low-intensity resistance training before high-intensity resistance training caused an increase in arterial stiffness, while high-intensity resistance training before low-intensity resistance training resulted in no change in arterial stiffness [20]. This can have many implications. One is that low-intensity resistance training can decrease arterial stiffness if done alone. Because low-intensity resistance training before high-intensity resistance training caused a significant increase in arterial stiffness, low-intensity resistance training negates the effects of high-intensity resistance training when done after it. This can also indicate that high-intensity resistance training increases arterial stiffness; however, this cannot be confirmed from one study alone.

Significant decreases in arterial stiffness

In our analysis, six out of the 23 papers reported a decrease in arterial stiffness after resistance training [23-28]. Okamoto et al. (2011) reported that high-intensity resistance training increased arterial stiffness and found that low-intensity resistance training decreased arterial stiffness [27]. However, in another paper published in 2017, a significant decrease in arterial stiffness was seen in both high-intensity and low-intensity resistance training [23]. This contradicts the previous finding that high-intensity resistance training increases arterial stiffness. A 2008 study also showed that resistance training decreases arterial stiffness if done through group circuit training twice a week for 12 weeks [26]. The once-a-week group saw no change in arterial stiffness [26]. Because the study sample included only elderly women, there is not enough information to state that the time between resistance training sessions is a factor in women of all age groups or of men at all. The difference in arterial stiffness values between the once-a-week and twice-a-week groups can also be due to the number of resistance training sessions that were done; hence, potentially having a group perform the training twice every other week can be a good control to confirm the finding. In the study by Robinson et al., a lower-body leg press was used as a resistance exercise along with a pre-workout supplement. The study reported a decrease in arterial stiffness in young healthy adults [28]. This is different from another study where lower body exercises caused no change in arterial stiffness [19]. However, there was a clear decrease in arterial stiffness which was not attributed to the pre-workout supplement in the study by Robinson et al.. The authors found that the study by Robinson et al. reported no impact on arterial stiffness by pre-workout supplements. In another study, there were decreases in peripheral arterial stiffness in prehypertensive patients after a resistance exercise [24]. However, another study reported an increase in central arterial stiffness of prehypertensive and hypertensive patients [16]. This indicates that peripheral and central arterial stiffness are affected differently by resistance exercise, implying that there is even more nuance to the finding that arterial stiffness increases with resistance training. Finally, in one study, patients with kidney transplants in the resistance training group showed a decrease in arterial stiffness which was even greater than the group performing aerobic training [25]. This could indicate that the kidneys are an essential organ for arterial stiffness, and potentially, there is a different effect of resistance training on patients with kidney transplants.

No significant changes in arterial stiffness

In our review, 10 papers out of the 23 showed no significant changes to arterial stiffness as a result of resistance training [29-38]. Three papers showed no significant change to arterial stiffness after testing overweight and obese adults [31,33,35]. This indicates that the impact of resistance training on the arterial stiffness of obese adults is not as great, or the resistance training performed was not effective at changing arterial stiffness values, which could be the same with many of the studies showing no change in arterial stiffness. Another study tested the effect of low-intensity resistance training and showed no change in arterial stiffness among obese postmenopausal women [34]. However, there was a decrease in arterial stiffness in the group that consumed a hypocaloric diet with resistance training [34]. This can strengthen the argument that resistance training does not affect the arterial stiffness of obese or overweight individuals. However, the study also showed that a hypocaloric diet plays a role in reducing arterial stiffness. Another study showed that a high-fat diet with resistance training decreases peripheral arterial stiffness with no effect on central arterial stiffness [29]. However, when the high-fat diet was consumed without resistance training, there was an increase in arterial stiffness [29]. Thus, a high-fat diet changes the way resistance training impacts peripheral arterial stiffness. There was again a difference in the way resistance training impacted central and peripheral arterial stiffness; however, the study posed the possibility that diet composition can impact this interaction. One study showed no change in arterial stiffness in middle-aged women after a bout of moderate-intensity resistance training [38]. Therefore, if high-intensity resistance training increases arterial stiffness and low-intensity resistance training decreases arterial stiffness [27], it is logical to presume that moderate-intensity resistance training causes no significant change in arterial stiffness. However, we cannot use the data from this study to conclude that moderate-intensity resistance training does not decrease the incidence rates of CVD, and further studies are needed to prove this hypothesis. Table 1 lists the arterial stiffness findings reported in the randomized controlled trials included in this review.

**Table 1:** Arterial stiffness reported in multiple randomized controlled trials.

Author (year)	Arterial stiffness before training (pulse wave velocity in m/s)	Arterial stiffness after training (pulse wave velocity in m/s)	Trend of arterial stiffness change
Au et al. (2017) [23]	6.24	5.77	Significant decrease
Augustine et al. (2014) [29]	5.4	5.6	No significant change
Beck et al. (2013) [24]	8.63	7.71	Significant decrease
Collier et al. (2008) [16]	11	12.7	Significant increase
Craighead et al. (2021) [30]	9.43	9.64	No significant change
Croymans et al. (2013) [31]	6.7	6.6	No significant change
DeVallance et al. (2016) [32]	6.9	7.0	No significant change
Fernandez-del-Valle et al. (2018) [33]	6.64	6.74	No significant change
Figueroa et al. (2013) [34]	11.87	11.46	No significant change
Greenwood et al. (2015) [25]	9.1	7.7	Significant decrease
Jefferson et al. (2016) [35]	13.87	13.60	No significant change
Kujawski et al. (2018) [36]	10	10	No significant change
Lin et al. (2016) [37]	7.31	7.31	No significant change
Miura et al. (2008) [26]	15.982	14.731	Significant decrease
Okamoto et al. (2006) [17]	9.9	10.9	Significant increase
Okamoto et al. (2009) [18]	10.49	11.53	Significant increase
Okamoto et al. (2009) [19]	Upper limb: 11.21 Lower limb: 11.55	Upper limb: 12.66 Lower limb: 11.46	Significant increase in upper limb. No significant change in lower limb
Okamoto et al. (2011) [27]	10.93	10.2	Significant decrease
Okamoto et al. (2013) [20]	6.1	7.4	Significant increase
Okamoto et al. (2015) [21]	7.1	7.6	Significant increase
Palmiere et al. (2018) [22]	5	5.5	Significant increase
Robinson et al. (2018) [28]	6.2	4.9	Significant decrease
Yoshizawa et al. (2009) [38]	9.2	9	No significant change

## Conclusions

The conclusions drawn from this study challenge the current understanding of the effect of resistance training on arterial stiffness. The analysis of the 23 randomized controlled trials found that the majority of the studies claim that resistance training has no significant impact on arterial stiffness. However, it is important to acknowledge that multiple conclusions were drawn from this meta-analysis. The type of resistance training performed had an effect on the central pulse wave velocity values. Those that recorded arterial stiffness values found major differences while training distinct parts of the body, leading to concerns regarding the reliability of the study. Some studies indicated that hypertension may have either led to an increase in arterial stiffness or resulted in no change. Moreover, the order in which the resistance training is performed can also impact arterial stiffness. Although a few studies reported that resistance training did not have an effect on the arterial stiffness of obese individuals, some claimed that diet composition can affect central and peripheral arterial stiffness. A hypocaloric diet was found to reduce arterial stiffness, and a high-fat diet with resistance training decreased peripheral arterial stiffness and increased it when there was no training involved. The current data will allow future researchers to identify various factors (the resistance protocol, training different parts of the body, obesity, cholesterol levels, diet consumption, and the type/order of training) that need to be considered when determining the effect of resistance training on arterial stiffness. Additionally, future investigations should also include a larger sample size that takes racial and ethnic variation of the participants into account before drawing any conclusions. Considering that a loss of arterial stiffness is a major factor in CVDs, this data can be beneficial in developing new and advanced treatment plans that involve resistance training.
